# Investigation of dental elastomers as oral mucosa simulant materials

**DOI:** 10.1002/cre2.399

**Published:** 2021-01-29

**Authors:** Joanne Jung Eun Choi, Shiyao Chen, John Neil Waddell

**Affiliations:** ^1^ Faculty of Dentistry Sir John Walsh Research Institute, University of Otago Dunedin New Zealand

**Keywords:** dental elastomer, mechanical properties, oral mucosa, silicone

## Abstract

**Objective:**

To measure mechanical properties of dental soft liners in tensional stress to identify their suitability as human oral mucosa simulant materials.

**Methods:**

Eleven different dental elastomers were subjected to tensile testing to obtain their tensile strength and elastic moduli (*n* = 15/group) according to the ISO‐527 method. Fractured surfaces of one specimen per sample group were examined under the light microscope and scanning electron microscope (SEM). Energy‐dispersive X‐ray spectroscopy (EDS) was performed for the elemental analysis or chemical characterization of each sample group. The obtained data were quantitatively and qualitatively analysed. They were also statistically analysed using SPSS version 25.

**Results:**

The tensile strength of dental elastomers ranged from 0.43 MPa (±0.09) to 7.41 MPa (±1.11) and had statistically significant differences between tested groups (*p* = 0.001). Vertex soft heat‐cure soft liner, GC impression silicones and Silagum soft liners were found to have tensile strengths close to that of the oral mucosa reported by previous studies. SEM analysis revealed that the elastomers with higher filler contents showed rough fractured surface with plucking of particles after tensile fracture.

**Conclusion:**

This is the first study assessing the suitability of dental elastomers as human oral mucosa simulant materials which can be used for in vitro, mathematical modeling and finite element analysis (FEA) to study masticatory force distribution in oral mucosa. Out of 11 studied, six (Vertex Soft, GC heavy and Light body, Molloplast B, Algin X Ultra and Exaclear) dental elastomers showed similar mechanical properties to the Theil embalmed gingival tissues. Vertex Soft, GC Light body, and Molloplast B may be used for the majority of oral mucosal model when considering tensile strength as the primary factor for mechanical stimulation.

## 1 INTRODUCTION

Trend regards to increasing shift of global ageing population is forthcoming especially in developed nations (UN, 2016), resulting in an increase of edentulous demographic and the demand of prosthodontic treatments (Boucher, 2004). Prosthodontic appliances including complete and partial dentures must rest upon the mucoperiosteum of the residual ridge and palate of the patient for retentive support (Tanaka et al., 2004). During mastication, the oral mucosa exhibits complex biomechanical behavior due to variations in cellular and extracellular compositions that behaves independently upon different physiological stresses (Kydd & Daly, 1982). These tissues are subjected to a wide variant of mechanical forces, including hydrodynamic forces, compression, elongation, friction, and shear generated during mastication (Chen et al., 2015). Despite advances in dental materials and techniques implemented during the manufacturing of removable complete dentures for more accurate fit of the denture, edentulous patients frequently experience pain and discomfort in the oral mucosa, in turn preventing prolonged use of their prosthodontic appliance (Kydd & Daly, 1982; Tanaka et al., 2004). Thus the lining oral mucosa of an edentulous patient possess a substantial role with denture‐supporting tissue interface and occlusal load distribution towards the underlying bone structure (Chen et al., 2015).

Mechanically, the oral mucosa is found to behave as viscoelastic material that demonstrates distinct time‐dependent properties upon loading (Tanaka et al., 2004). At present the histological nature of oral mucosa is well established (Avery et al., 2002; Groeger & Meyle, 2019), however the biomechanical properties can alternate vastly across the literatures (Kydd & Mandley, 1967; Lytle, 1962). Since the 1970s, researchers have been studying the mechanical properties of the oral mucosa in areas regarding its elasticity, viscosity and permeability (Chen et al., 2015; Goktas et al., 2011; Kydd & Daly, 1982; Kydd & Mandley, 1967; Sawada et al., 2011). Earlier studies have modeled the elastic properties of the oral mucosa to be linear, meaning the materials have a straight stress–strain response curve with a constant elastic modulus (Chen et al., 2015). Using dead weight or instant loading, these earlier investigations discovered a wide range of elastic modulus ranging from 0.88 to 11.12 MPa and compressive modulus around 1.10 to 3.90 MPa (Kydd & Daly, 1982). Kydd and Mandley (1967) found the mucosa is generally stiffer under tension than compression (Kydd & Mandley, 1967). Further research realized the biomechanical response of the oral mucosa undergoing hyper‐elasticity, applied in the mechanics of rubber‐like materials that behaves exactly like biological soft tissue under both normal and pathological conditions and showing a viscoelastic time‐dependent response property (Chen et al., 2015; Kydd & Mandley, 1967; Lytle, 1962). Studies carried out in vivo on viscoelasticity of human oral mucosa vary greatly compared to in vitro studies 0.08 MPa ± 0.03 (Sawada et al., 2011). Several earlier in vitro studies used porcine and monkey oral mucosa as a substitute, confirming the stress and location of oral mucosa dictating its elastic modulus, ranging from 2.48 to 19.75 MPa (Goktas et al., 2011; Inoue et al., 1985; Lacoste‐Ferré et al., 2011). However, inaccuracies have been identified within the reported values due to the miscalculation in the conversions of the unit presented (Chen et al., 2015). Despite interests in the finding of stress distribution within the oral environment with types of denture restorations, multiple studies disregard to identify the legitimate origin on the mechanical properties of mucosa (either hard, resilient, or soft), leading into study that such as Finite Element Analysis (FEA) to be conducted with false statistics on the mechanical properties of the human oral mucosa (Bacchi et al., 2012; Barão et al., 2008; Dos Santos et al., 2011; Ko et al., 1992).

Simulation of oral mucosa can result in multiple biomechanical behaviors that are closely decisive in clinical application, especially for the function in distributing masticatory forces (Maruo et al., 2010; Żmudzki et al., 2015). Thus, it is important to explore the possibility of an oral mucosal simulant applicable to a practical biomechanical model, with existent dental materials, that can serve the purpose to interpret, analyze and predict the cumulative biomechanical behavior of the mucosa in response to dental prostheses and optimize treatment outcome.

The authors' previous study (Choi et al., 2020) had identified the tensile strength and elastic modulus of two Thiel‐embalmed cadavers to be location‐dependent and ranging from 37.36 MPa ± 17.4 in the attached gingiva group, followed by samples from the hard palate (18.13 MPa ± 4.5) and buccal mucosa (8.33 MPa ± 5.8), which has been identified closest resemblance possible to the human oral mucosa. Defining the mechanical characterization of living human oral tissues has been difficult, due to the ethical issues and difficulties of sourcing tissue samples for testing (Choi et al., 2020; Ottone et al., 2016; Thiel, 1992). Fresh human cadaveric tissues are of extremely limited supply for biomechanical testing and they start to deteriorate rapidly with a potential risk for infection (Choi et al., 2020; Ottone et al., 2016; Thiel, 1992). This leads to the investigation of simulant materials of oral mucosa to build physical simulation models, however, to date there is no study available to investigate the simulant materials for oral mucosa. Various dental impression silicone materials and denture soft liners are available on the market (Boucher, 2004; Sawada et al., 2011; Tanaka et al., 2004). Denture soft liners in particular, are known to have similar mechanical properties as the oral mucosa to dissipate the pressure exerted from the denture onto the mucosa, however, this has not been scientifically proven.

Therefore, the objectives of this study were to measure the mechanical properties of dental soft liners, in tensional stress and to identify their suitability as human oral mucosa simulant materials. The null hypothesis was that there is a dental silicone material that has similar mechanical properties as human oral mucosa.

## 2 MATERIALS AND METHODS

The compositions and manufacturer's information of 11 dental elastomers (dental impression silicones and denture soft liners) are summarized in Table 1. The materials were included in the current study due to their popularity and availability.

**TABLE 1:** Summary of dental elastomers used in this study

		Material name	Brand (Manufacturer)	Composition
Dental Impression material (Self‐cure)		Light Body	EHAHIFLEX Regular Type (GC DENTAL PRODUCTS CORP.)	Polyethylene glycol derivative 5%–10% methylhydrogen dimethylpolysiloxane 1%–5%
		Medium Body	EHAHIFLEX Injection Type (GC DENTAL PRODUCTS CORP.)	Polyethylene glycol derivative 5%–10% methylhydrogen dimethylpolysiloxane 1%–5%
		Heavy Body	EHAHIFLEX Tray Type (GC DENTAL PRODUCTS CORP.)	Polyethylene glycol derivative 5%–10% methylhydrogen dimethylpolysiloxane 1%–5%
		Algin X Ultra	Dentsply Sirona	Cristobalite <30% Diatomaceous earth, flux‐calcinated <20% Silica amorphous, <5% Titanium dioxide <3%
		Exaclear	GC DENTAL PRODUCTS CORP.	Clear vinyl polysiloxane
Dental Soft liner	Self Cure	GC Soft‐liner	GC DENTAL PRODUCTS CORP.	PMMA and ethyl alcohol liquid
		Silagum	DMG Chemical Pharmaceutical Factory GmbH	Vinyl polysiloxane
		Ufi Gel SC	VOCO GmbH	Vinyl polysiloxane
	Heat Cure	Vertex Soft	Vertex Dental	PMMA
		Molloplast B	DETAX	dibenzoyl peroxide; benzoyl peroxide; Dodecaemthylcyclohexasiloxane
	3D Printed	DentaGum	ASIGA	7,7,9(or 7,9,9)‐ trimethyl‐4,13‐dioxo‐ 3,14‐dioxa‐5,12‐ diazahexadecane‐1,16‐ diyl bismethacrylate 10%–25% Tetrahydrofurfuryl methacrylate 10%–20% Diphenyl(2,4,6‐ trimethylbenzoyl) phosphine oxide 10%–20%

**FIGURE 1:**
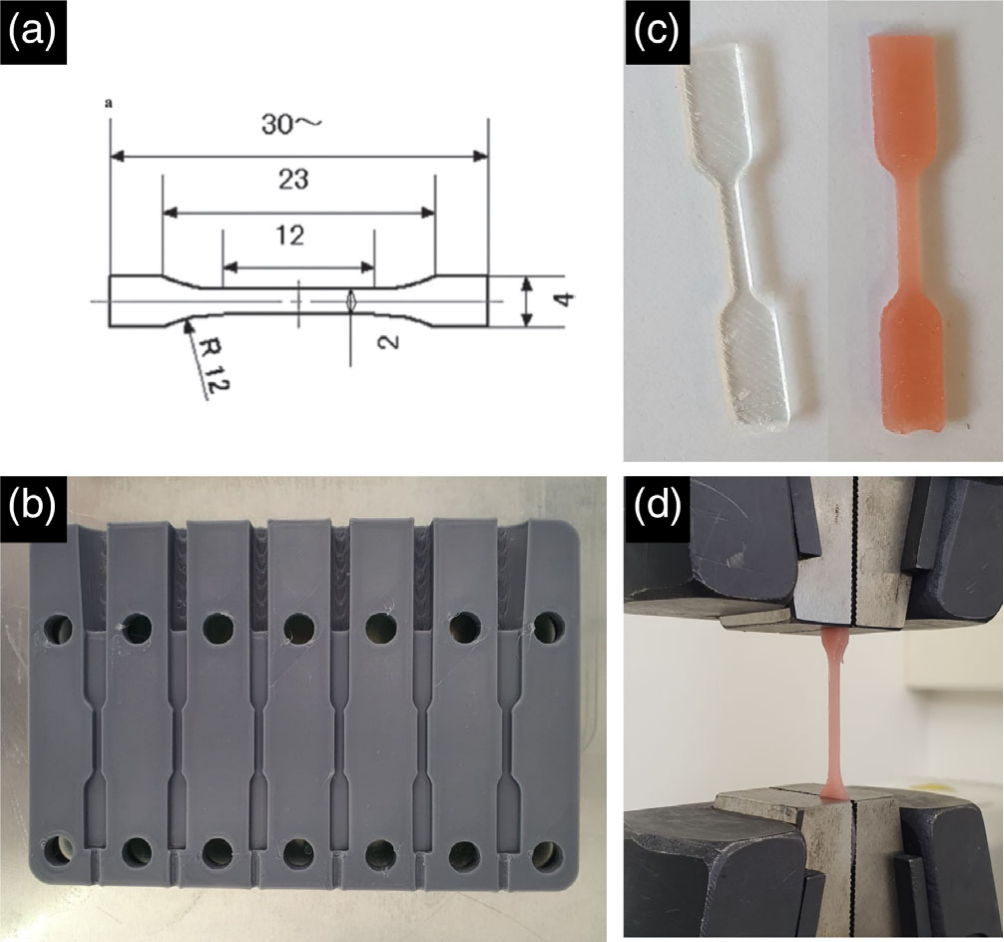
Images showing (a) specimen dimension for tensile testing (modified from ISO517), (b) customized jig used to produce specimens, (c) examples of the testing specimens, (d) specimen under tension for testing

The geometry of the specimens were type 1B according to EN ISO 527‐1 (Figure 1). The self‐curing specimens were fabricated by injecting the silicone material from the manufacturer's designated automated dispensing gun into a 3D‐printed mold (Form 2, Formlabs) allowing for easy demoulding. The heat‐curing specimens were fabricated in dental stone molds created from a 3D printed pattern and heat cured according to the manufacturer's instruction. For the 3D printed gum resin (DentaGum, Asiga), a STL file of the dumbbell shaped specimen was constructed via AutoCAD (Autodesk), and specimens were printed in an Asiga 3D printer (Asiga Max, Asiga). The printing and post curing process were done as per manufacturer's instructions.

**FIGURE 2:**
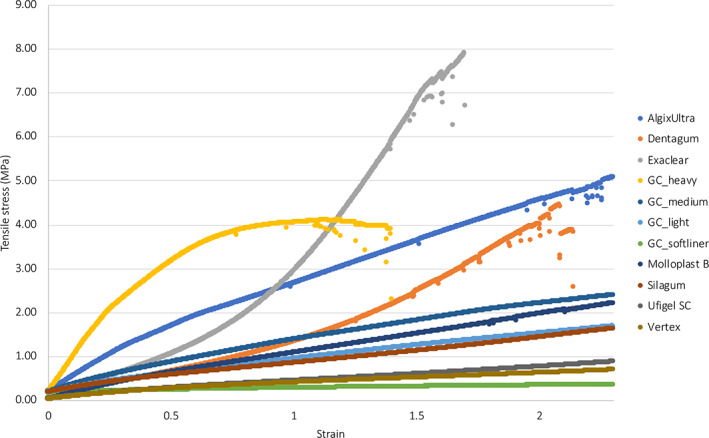
Graph showing the stress and strain curve of 11 materials investigated up to a strain rate of 2.5

**FIGURE 3:**
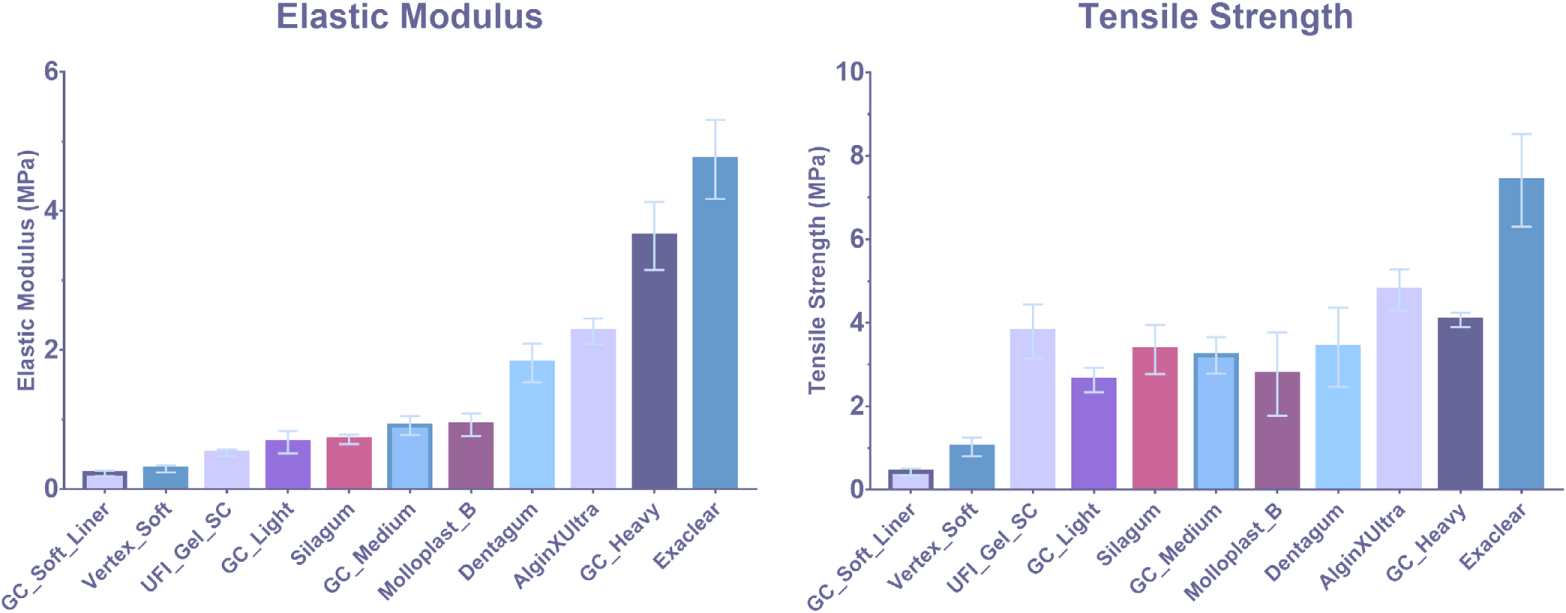
Tensile testing of all sample groups when comparing both E‐modulus and tensile strength

Tensile testing was performed according to EN ISO 527‐1. Flat dumbbell shaped specimens (*n* = 15/group; total *n* = 150) were loaded in tension along their longitudinal axis in a universal testing machine (Instron 3369; Instron), using a 1 kN (±2) load cell at a constant speed of 1 mm/min with an extensometer (W‐6280 series, Instron) used to record the strain until failure occurred. The maximum force (N), tensile stress (MPa) and strain (mm) were recorded. To obtain E modulus in tension for the silicones, ETAG‐002 refers to ISO 527‐1, which postulates that this parameter can be calculate according to Equation (1).

(1) $$E=\frac{\sigma_{2}-\sigma_{1}}{\varepsilon_{1}-\varepsilon_{2}},$$

where E = young's modulus, σ_1_ = the stress in MPa as measured on the deformation value of ε_1_ = 0.05, σ_2_ = the stress in MPa measured on the deformation value of ε_2_ = 0.025. The mean values of tensile strength and E modulus of each group are presented in Table 2 and Figures 2 and 3. The statistical analysis was performed using ANOVA via SPSS Version 25 (IBM) and the level of significance was set at *p* < 0.05.

**TABLE 2:** Mechanical properties of dental elastomers versus Thiel‐embalmed and porcine oral tissues (MPa ± SD)

	Failure load (N)	Tensile strength (MPa)	E‐modulus (MPa)	Oral mucosa tensile strength (MPa ± SD)			Oral mucosa E‐modulus (MPa)	Porcine oral soft tissue		
				Gingiva (3.81 ± 0.94)	Hard palate (1.70 ± 0.87)	Buccal mucosa (1.54 ± 0.52)		Failure load	Tensile strength	E‐modulus
GC Soft Liner	1.19 ± 0.25	0.43 ± 0.09	0.22 ± 0.04							
Vertex Soft	4.06 ± 0.8	1.03 ± 0.23	0.29 ± 0.38		✔	✔			b,c	
GC Light	6.32 ± 1.36	2.35 ± 0.75	0.66 ± 0.16	✔	✔	✔			a,d,e	
Molloplast B	11.39 ± 3.65	2.77 ± 1.00	0.89 ± 0.16	✔	✔	✔		a,b,c,d	a,d,e	
GC Medium	8.21 ± 0.87	3.22 ± 0.43	0.90 ± 0.13	✔				a,b,c,d	d,e	
Silagum	8.92 ± 1.16	3.36 ± 0.59	0.71 ± 0.07	✔				a,b,c,d	d,e	
Dentagum	10.24 ± 2.84	3.41 ± 0.95	1.84 ± 0.29	✔				a,b,c,d	d,e	
UFI Gel SC	9.53 ± 2.64	3.54 ± 0.95	0.58 ± 0.10	✔				a,b,c,d	d,e	
GC Heavy	10.49 ± 0.3	4.08 ± 0.17	3.58 ± 0.47	✔			Buccal Mucosa (8.33 ± 5.78)	a,b,c,d	e	a
Algin X Ultra	12.7 ± 1.8	4.65 ± 0.72	2.73 ± 0.19	✔				a,b,c,d	e	a,c
Exaclear	19.91 ± 2.14	7.41 ± 1.11	4.75 ± 0.57					e		a,b

*Note*: Summary data when comparing failure load, tensile strength, and elastic modulus of selected dental elastomers when compared to Thiel‐embalmed edentulous human oral mucosa and porcine oral soft tissues. For porcine oral soft tissues, representative letters indicate various soft tissues location and their respective mechanical properties: (a) is lingual alveolar mucosa, (b) buccal alveolar mucosa, (c) is buccal mucosa, (d) lingual attached gingiva, and (e) is buccal attached gingiva.

All fractured specimens were analysed under the light microscope (C‐DSS230, Nikon) at x40 and x60 magnifications and a representative specimen from each group was examined by Scanning Electron Microscopy at x 300 magnification (SEM, Zeiss Sigma VP, Zeiss) and Energy‐dispersive X‐ray spectroscopy (EDS, Zeiss Sigma VP, Zeiss) was performed for the elemental analysis and chemical characterization of a specimen. The obtained data were quantitatively and qualitatively analysed.

## 3 RESULTS

Tensile testing measurements conducted on the dental elastomer materials are identified to possess a great range of tensile strength and elastic modulus, although the magnitude of both determinants does not seem to have a direct relation with each other. From Choi et al. (2020)'s study, the Thiel‐embalmed gingiva's tensile strength was found to be 3.81 ± 0.94 MPa whereas that of the porcine gingiva found by Goktas et al. (2011) study was 3.94 ± 1.19 MPa (Choi et al., 2020; Goktas et al., 2011). Illustrated in Table 2, GC Heavy body, Algin X Ultra, and Exaclear all presented similar elastic modulus within the range of Theil‐embalmed buccal oral mucosa (8.33 ± 5.78 MPa), comparable to the porcine lingual alveolar, buccal alveolar, and buccal mucosa. Although those materials were compatible, One‐way ANOVA analysis shows statistical significant differences between them with *p*‐value of <0.001, alongside incremental decrease in elastic modulus from Exaclear to Algin X Ultra the GC Heavy body (4.75 ± 0.57, 2.73 ± 0.19, and 3.58 ± 0.47 MPa). With significant difference between each material, additional interpretation can be identified when comparing with porcine oral mucosa, where Exaclear fall within buccal alveolar mucosa, Algin X Ultra is comparable to buccal mucosa, and lingual alveolar matches to all three materials. When comparing the two graphs in Figure 3, there seems to be no direct relations between the tensile strength and elastic modulus of a material when ranked using E‐modulus (Figure 3). However, the highest and lowest of elastic modulus and tensile strength are identified as consistent within the sample groups, namely SC Soft Liner, Vertex Soft and Exaclear. For elastic modulus statistically significant finding was observed between almost the entire sample groups ranging from *p*‐value <0.001 to *p*‐value <0.046. Statistical significance was also present among the sample groups for tensile strength.

In preference, tensile strength data suggests the possibility of several or one dental elastomer that have similar properties to all mucosa types found within the oral environment. When comparing tensile strength, Vertex soft denture lining material was 1.03 ± 0.23 MPa and was in agreement to the tensile strength value found for Theil‐embalmed hard palate (1.70 ± 0.87 MPa) and buccal mucosa (1.54 ± 0.52 MPa), as well as porcine buccal alveolar and buccal mucosa (Choi et al., 2020; Goktas et al., 2011). In addition to Thiel‐embalmed hard palate and buccal mucosa, GC light body (2.35 ± 0.75 MPa) and Mollopast B (2.77 ± 1 MPa) both acquire suitable tensile strength value to Thiel‐embalmed gingiva tissue (3.81 ± 0.94 MPa) and porcine lingual alveolar mucosa, lingual attached gingiva and buccal attached gingiva (Goktas et al., 2011). No statically significant difference was calculated between GC light body and Molloplast B (*p*‐value >0.99), whereas significant difference was present in Vertex Soft when comparing to GC light body and Molloplast B (*p*‐value <0.001).

Figure 2 shows the stress and strain curves of 11 materials investigated in the study. GC‐heavy body material and Algin‐X Ultra show a typical viscoelastic behavior of dental elastomers, whereas Exaclear and DentaGum's stress and strain curves show a typical stiff hardening as the materials underwent tension. The other materials show a linear stress–strain type curve.

**FIGURE 4:**
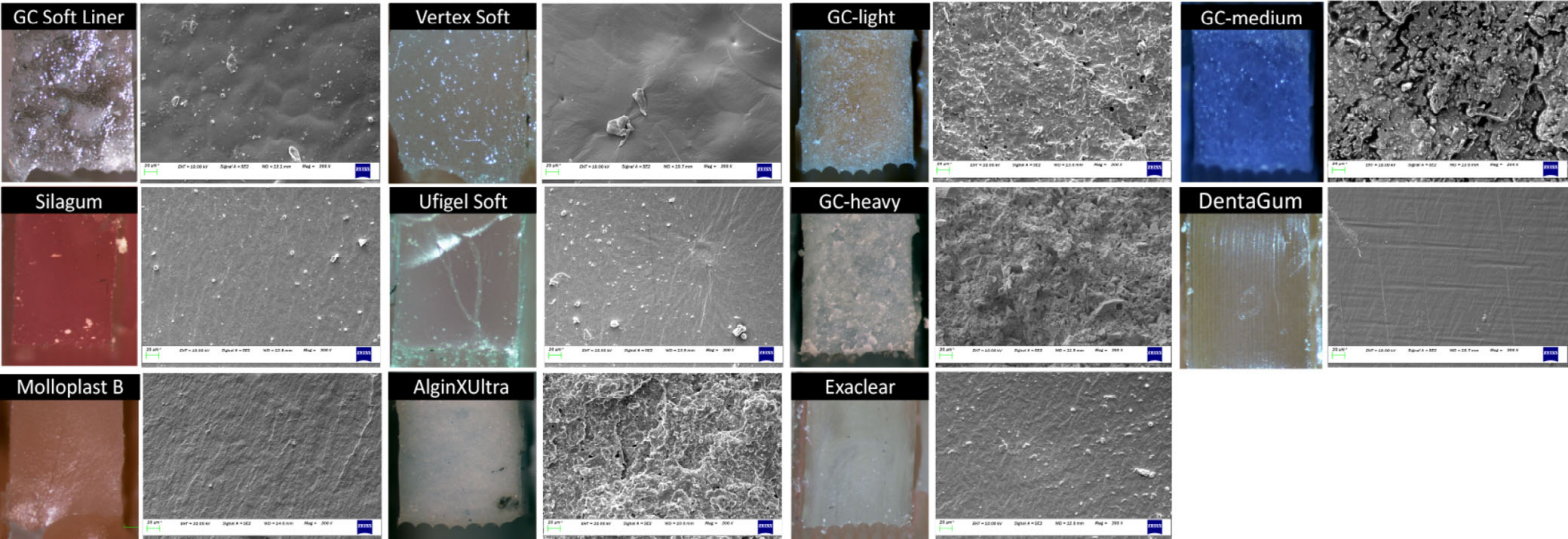
Light microscope and scanning electron microscope (SEM) images of each group at fractured interface

Light microscope and scanning electron microscope (SEM) analysis revealed that GC soft liner and Vertex soft which showed the lowest tensile strength had a clean fractured surface under tension, whereas the materials which showed higher tensile strengths such as GC‐light, medium and heavy body impression materials, Molloplast B and Algin‐X Ultra showed rough, irregular fracture surfaces (Figure 4). Silagum and Ufigel soft materials had a relatively smooth fractured surface with irregular particles observed on the surface. DentaGum showed a smooth fracture surface with linear indents all over the surface. EDS analysis revealed that all 11 materials had a high content of Carbon 39.5%–83.5%. All materials, except DentaGum and Vertex Heat cured denture lining material, presented with varying amounts of silica content, ranging from 0.2% to 28.4%. The Algin‐X Ultra, GC Exhaiflex medium body and GC Exhaiflex heavy body materials, which showed the roughest fracture surfaces, were found to contain low percentages of elements such as aluminium, titanium and sodium (Table 3 and Figure 4).

**TABLE 3:** Summary of EDS results showing the chemical composition of the materials tested

Materials	Chemical composition (%)
Ufigel SC	C 44%, Si 28.4%, O 27.7%
Algin X ultra	C 45.9%, Si 22.8%, O 31.2%, AL + Ti + Na
GC soft liner	C 83.5%, Si 0.2%, O 16.4%
Silagum	C 81.3%, Si 0.3%, O 18.4%
Exaclear	C 48.1%, Si 25.1%, O 26.8%
Molloplast B	C 47.2%, Si 27.1%, O 25.7%
GC Exhaiflex (medium body)	C 49.5%, Si 24.6%, O 24.6%, Ai 0.2%
GC Exhaiflex (heavy body)	C 39.5%, Si 24.8%, O 35.3%, Ti Na CI Al
GC Exhaiflex (light body)	C 47.8%, Si 27.5%z, O 24.7%
Dentagum 3D printed	C 74.7%, O 25.3%
Vertex heat cure	C 79.8%, O 20.2%

## 4 DISCUSSION

Simulation of oral mucosa can result in multiple biomechanical behaviors that are closely decisive in clinical application, especially for the function in distributing masticatory forces. Therefore the current study measured the mechanical properties of 11 different elastomers in tensional stress to identify their suitability as human oral mucosa simulant materials. The null hypothesis was accepted due to several selected materials were found to be compatible with the mechanical properties of the Theil‐embalmed or porcine oral mucosa tissues (Choi et al., 2020; Goktas et al., 2011).

Dental elastomer (silicone) is one of the widely used dental materials due to its unique properties, including biocompatibility, superior temperature, chemical and aging resistance (Feng et al., 2017; Ueno et al., 2011). The dental silicone's viscoelastic recovery rates and the effect of inclusion of filler particles on strength have been previously studied, however, there were only limited reports investigating its tensile and elastic properties (Kang, 2001; McCabe & Carrick, 1990; Wieckiewicz et al., 2016).

Identifying materials that simulate the mechanical properties of oral mucosa has numerous advantages; it enables the building of a physical/anatomical model of the human oral cavity, the jaw with soft tissues which can provide a wide range of information (Isobe et al., 2013; Schwarz, 1992). For example, Sato et al. (2020) recently reported an in vitro study investigating the pressures exerted onto the soft tissues underneath different types of implant overdentures (Sato et al., 2020). Although their study showed a valid comparison between pressure distribution, the in vitro testing was conducted on a conventional plastic simulation jaw model which has a silicone lining to act as the oral mucosa on the model. However, there is no scientific evidence that the silicone lining used was showing a similar behavior to the human oral mucosa, questioning the validity of the study. For this reason, previous studies used mathematical modeling and FEA (Chen et al., 2015; Ueno et al., 2011), however there is no strong evidence that the mechanical properties data used for the modeling was accurate, and again the validity of the results may be questionable.

Through the use of dental material that serves the purpose of tissue conditioning and behaves in a viscoelastic or elastic manner to impede the traumatic forces during mastication, this study included 11 commonly used dental silicones (dental impression material and denture soft liners), to identify which one mimics most closely to the mechanical properties of oral mucosa. In the course of applied functional load, often the edentulous surface experiences a consolidation of mechanical stress (e.g., compression, tensile, and shear forces) along difference directions (Chen et al., 2015). In order for consistent and accurate outcome, tensile testing via universal testing machines was employed to minimize variables as well as reproduction of previous correlating studies (Choi et al., 2020; Goktas et al., 2011). Out of 11 dental elastomers studied, six silicones, including Vertex Soft, GC Light body, Molloplast B, GC Heavy body, Algin X Ultra, and Exaclear have similar mechanical properties in either elastic modulus or tensile strength to the associated oral mucosal tissues (Choi et al., 2020; Goktas et al., 2011). Vertex Soft is found to have the most similar tensile strength to the hard palate and buccal mucosa tissues, whereas the other silicones (GC light body and Molloplast B) studied were compatible with all three types of oral tissues (gingiva, hard palate, and buccal mucosa), and due to the highly variant gingival tissues many other elastomers (total 9, Figure 3) fit within the range therefore can be considered supplementary depending on the desired material (Choi et al., 2020; Goktas et al., 2011). Previous investigation upon the elastic modulus of Thiel‐embalmed human oral mucosa and porcine oral tissues concluded highly variant values alongside low tensile stress, ranging from gingiva tissue (37.36 ± 17.36 MPa) to buccal mucosa (8.33 ± 5.78 MPa) and for porcine lingual attached gingiva has elastic modulus of 18.83 ± 5.98 MPa to lowest buccal mucosa 2.48 ± 0.37 MPa, implying the deformative nature of oral mucosa fluctuates especially among attached gingiva tissues (Choi et al., 2020; Goktas et al., 2011). Hence, the materials used in this study has only found to be compatible with mucosal tissue with lower elastic modulus such as buccal mucosa (8.33 ± 5.78 MPa) and alveolar mucosa, illustrated in Table 2.

Collected data suggests tensile strength and elastic modulus of silicone rubber is found to be low, since a small stress in the silicone rubber immediately causes a large deformation, which is also the case in human oral mucosa. The current study revealed that the tensile strength of 11 dental elastomers ranged from 0.40 MPa (±0.08) to 6.64 MPa (±0.71) and had statistically significant differences between tested groups (*p* = 0.001) and elastic modulus of 0.81 ± 0.78 and 20.99 ± 2.13 MPa. This was in agreement with the values reported by Inoue et al. (1985) of 0.66 MPa (Inoue et al., 1985) and 19.6 MPa reported by Takayama et al. (2001). Study of tensile stress analysis conducted on porcine oral soft tissue by Gokatas and colleagues, shown similar failure load and young's modulus with dental silicones, namely Exaclear and GC heavy body with associated lingual (Failure load of 10.54 ± 2.18 N, tensile strength of 1.72 ± 0.51 MPa, and elastic modulus of 4.79 ± 2.54 MPa) and buccal alveolar mucosa (Failure load of 8.93 ± 2.06 N; tensile strength of 1.29 ± 0.19 MPa; and elastic modulus of 5.74 ± 1.15 MPa). Similar trends with the stress–strain graph can also be identified with the corresponding materials, as most tested materials behave fully elastic prior to failure and in case of GC Heavy body illustrates substantial plastic deformation before failure.

Keratinisation of mucosa plays an important factor to the biomechanical properties of the tissue, where keratinized gingiva has increased tensile strength and stiffness relative to non‐keratinized mucosal regions (Goktas et al., 2011). Similar to filler particles in elastomers (Wieckiewicz et al., 2016), densely arranged elastin or collagen fibers contribute to mucosa with higher viscoelastic properties. Investigation upon width of keratinised mucosa for patients (*n* = 27) with implant supporting overdentures, shown to have an average keratinised mucosa width of 2.5 ± 1.5 mm ranging from 0–6 mm (Adibrad et al., 2009). This could potentially explain the exceedingly great variation in mechanical properties especially in elastic modulus from previous studies (Choi et al., 2020; Goktas et al., 2011). This further suggest that majority of mucosal tissue that occupies the edentulous surface is consisted of mostly non‐keratinized mucosa. When comparing to previous literature, these tissues are identified as buccal mucosa (Choi et al., 2020), lingual or buccal alveolar mucosa and buccal mucosa (Goktas et al., 2011). Establishing the fact that keratinised mucosa only plays a limited function throughout the load distribution of masticatory forces, eventual composition of a oral biomechanical model is therefore consisted of materials that could simulate buccal mucosal tissue which this study had identified 6 potential dental elastomers (Vertex Soft, GC Light body, Molloplast B, GC Heavy body, Algin X Ultra, and Exaclear) that feature both elastic modulus and tensile strength.

An interesting finding from the current study was that, although a similar ranking of tensile strength and elastic modulus values were present in Thiel‐embalmed tissues and porcine oral mucosa tissues, there was no rank correlation found between the tensile and elastic modulus values of the 11 dental silicones tested. This can be explained by the moduli of elastomers being influenced by the absence or amount of filler particles included in their formulation by the manufacturers to alter their properties and this is further influenced by the size, distribution and shape of the particles (Kang, 2001; McCabe & Carrick, 1990; Wieckiewicz et al., 2016). As seen in Figure 4 SEM images of Vertex Soft and Dental Gum have smooth fractured surfaces under tension indicating the absence or minimal filler particle content. Meincke et al. (2016) reported that polymethyl methacrylate based elastomers have less affinity and less surface activity in comparison with other elastomers with filler contents (Meincke et al., 2016). Dental elastomers tested in the current study which contained fillers showed a higher tensile strength and rougher surface at the fracture interface under tension. The GC Exhaiflex impression material range (light, regular and heavy body) and Algin‐X Ultra material showed a rough surface under SEM with some filler particles being “plucked” from the opposing surface (Figure 4). This may indicate the lack of bonding between filler particles and base elastomer matrix (Klingender, 2008; Meincke et al., 2016). Meincke et al. (2016) reported this to be common in silicone materials; fillers must make intimate contact with the elastomer chains if they are going to contribute to reinforcement of the polymer. Fillers may be used for reinforcement or to increase the viscosity and hardness of the material to improve the properties of tensile strength up to a limit, as the amount of the load increases (Kang, 2001; McCabe & Carrick, 1990; Wieckiewicz et al., 2016). While the hardness values increase, the values of other properties, such as resilience, decrease (Klingender, 2008; Meincke et al., 2016). This may again support the trend of some elastomers having higher tensile strength while their elastic modulus is low, and vice versa for the rest of the materials.

The EDS results showed a correlation between those elastomers with rough surface and a high Si content (>22.8%), namely the Ufigel SC, Algin‐X Ultra, Exaclear, Molloplast B, GC Exhaiflex light, medium and heavy body (Table 3). This would indicate that the material used for the filler particles were either Si based amorphous glass and or crystalline Si such as cristobalite (Algin‐X Ultra, Table 1).

A limitation of this study is that the investigation of dental elastomers was confined to only the tensile strength and elastic modulus. This was done in order to compare the results to previous study on Theil embalmed oral mucosa (Choi et al., 2020) and porcine oral mucosa (Goktas et al., 2011). To more thoroughly validate the use of dental elastomers as oral mucosa simulant materials, additional tests need to be performed on other mechanical properties such as shore hardness, creep analysis, dynamic mechanical analysis, and tear strength. Another limitation of the current study was the low strain rate used. This was done to correlate with the strain rate used in the Theil‐embalmed and porcine mucosa study. In future, intermediate, or higher strain rates may provide better insight into the viscoelastic behavior of both Theil and dental elastomers, adding valuable information for the search of a simulant material for human oral mucosa.

Soft denture liners are widely used for denture wearers who complain of masticatory pain (Kydd & Daly, 1982; Tanaka et al., 2004). The patients who normally receive a soft denture reline have a thin and non‐resilient oral mucosa and/or severe alveolar resorption (Hayakawa et al., 1994; Kimoto et al., 2007). When functional forces are transmitted to the basal mucosa through a hard denture base during mastication, it may hurt the patient's underlying mucosa through pressure induced nerve pain (Kydd & Daly, 1982; Tanaka et al., 2004). The results of the current study can also be used to give guidelines for dental practitioners to select the most appropriate denture reline materials to suit the patient's individual mucosa conditions. However, this will require further investigation into human oral mucosa from different locations and conditions. Furthermore, the behavior after water storage could be studied as well as the alteration of the properties with time, since normally the human or porcine oral mucosa are tested after being stored in an appropriate medium to simulate oral mucosa with saliva. It would be of advantage to also investigate the stress–strain curves not only in tension as studied in this study, but also in shear and compression. Such additional information will contribute to an improved theoretical description of the real in‐service behavior of dental elastomeric materials. These values could then be used to produce more accurate mathematical modeling/FEA studies which will enhance the prediction of mechanical behavior under different scenarios in the dental context.

## 5 CONCLUSION

Within the limitations of the study it can be concluded that:

- Six (Vertex Soft, GC Light body, Molloplast B, GC Heavy body, Algin X Ultra and Exaclear) dental elastomers have similar mechanical properties to the Theil embalmed gingival tissues.
- Vertex Soft, GC Light body, and Molloplast B may be used for the majority of oral mucosal model when considering tensile strength as the primary factor for mechanical stimulation.
- In cases where simulation of patient with non‐keratinised epithelium biomechanical models, GC heavy body, Algin X Ultra, and Exaclear could be used to replicate the elastic modulus of buccal mucosa. However, to simulate keratinized epithelium or attached gingiva, that has higher collagen content thus increasing elastic modulus, more resilient material is required or modification of above‐mentioned elastomers such as increasing the filler particle ratio.

## AUTHOR CONTRIBUTIONS

**Joanne Jung Eun Choi:** conceptualization, data curation, formal analysis, investigation, methodology, writing original draft, writing–review and editing. **Shiyao Chen:** data curation, formal analysis, investigation, methodology, writing of original draft, writing–review and editing. **John Neil Waddell:** conceptualization, formal analysis, investigation, writing–review and editing.

## CONFLICT OF INTEREST

The authors declare no conflict of interest.

## Data Availability

The data that support the findings of this study are available from the corresponding author upon reasonable request.
